# Liver metastasis of pancreatic cancer: the hepatic microenvironment impacts differentiation and self-renewal capacity of pancreatic ductal epithelial cells

**DOI:** 10.18632/oncotarget.25884

**Published:** 2018-08-03

**Authors:** Hendrike Knaack, Lennart Lenk, Lisa-Marie Philipp, Lauritz Miarka, Sascha Rahn, Fabrice Viol, Charlotte Hauser, Jan-Hendrik Egberts, Jan-Paul Gundlach, Olga Will, Sanjay Tiwari, Wolfgang Mikulits, Udo Schumacher, Jan G. Hengstler, Susanne Sebens

**Affiliations:** ^1^ Institute for Experimental Cancer Research, Christian-Albrechts-University Kiel (CAU) and University Medical Center Schleswig-Holstein (UKSH) Campus Kiel, Kiel, Germany; ^2^ Department of Pediatrics, UKSH Campus Kiel, Kiel, Germany; ^3^ Department of Medicine I, University Medical Center Hamburg-Eppendorf, Hamburg, Germany; ^4^ Department of General, Visceral-, Thoracic-, Transplantation- and Pediatric Surgery, UKSH Campus Kiel, Kiel, Germany; ^5^ Molecular Imaging North Competence Center, Clinic of Radiology and Neuroradiology, CAU and UKSH Campus Kiel, Kiel, Germany; ^6^ Department of Medicine I, Division: Institute of Cancer Research, Comprehensive Cancer Center, Medical University of Vienna, Vienna, Austria; ^7^ Centre of Experimental Medicine, Department of Anatomy and Experimental Morphology, University Medical Centre Hamburg-Eppendorf, Hamburg, Germany; ^8^ Leibniz Research Centre for Working Environment and Human Factors (IfADo), Technical University Dortmund, Dortmund, Germany

**Keywords:** pancreatic ductal adenocarcinoma, cancer stem cell, EMT, epithelial-mesenchymal-transition, hepatic microenvironment

## Abstract

Pancreatic ductal adenocarcinoma (PDAC) is often diagnosed at advanced stages with the liver as the main site of metastases. The hepatic microenvironment has been shown to determine outgrowth of liver metastases. Cancer stem cells (CSCs) are essential for initiation and maintenance of tumors and acquisition of CSC-properties has been linked to Epithelial-Mesenchymal-Transition. Thus, this study aimed at elucidating whether and how the hepatic microenvironment impacts stemness and differentiation of disseminated pancreatic ductal epithelial cells (PDECs). Culture of premalignant H6c7-kras and malignant Panc1 PDECs together with hepatocytes and hepatic stellate cells (HSC) promoted self-renewal capacity of both PDEC lines. This was indicated by higher colony formation compared to cells cocultured with hepatocytes and hepatic myofibroblasts. Different Panc1 colony types derived from an HSC-enriched coculture were expanded and characterized revealing that holoclones exhibited an enhanced colony formation ability, elevated and exclusive expression of the CSC-marker Nestin and a more pronounced mesenchymal phenotype compared to paraclones. Moreover, Panc1 holoclone cells showed an increased tumorigenic potential *in vivo* leading to formation of undifferentiated tumors in 7/10 animals, while inoculation of paraclone cells only led to formation of tumors in 2/10 animals being smaller in number and size. Holoclone tumors were characterized by elevated expression of mesenchymal markers, complete loss of E-cadherin expression and high expression of Nestin. Finally, Etanercept-mediated TNF-α blocking partly reversed the mesenchymal CSC-phenotype of Panc1 holoclone cells. Overall, these data provide evidence that the hepatic microenvironment determines stemness and differentiation of PDECs, thereby substantially contributing to liver metastases of PDAC.

## INTRODUCTION

Pancreatic ductal adenocarcinoma (PDAC), the most common pancreatic neoplasia, is characterized by its particularly poor prognosis with a 5-year survival rate of only around 8% [1] and an ongoing increase in death rate [2]. Due to the lack of early and specific symptoms, the majority of patients presents with advanced or even metastasized disease which only leaves palliative treatment. The only curative option that is eligible to about 20% of patients is surgery and even after surgery, the 5-year survival is poor owing to recurring or metastatic tumor growth [3, 4].

The early occurrence of distant metastases even after removal of the primary tumor supports the idea of epithelial/tumor cell dissemination as an early event during malignant progression [5]. As a prerequisite for dissemination, epithelial/tumor cells undergo Epithelial-Mesenchymal-Transition (EMT) leading to the acquisition of a more motile and invasive phenotype. This is associated with reduced expression of epithelial proteins like E-cadherin and upregulation of mesenchymal markers such as N-cadherin, Vimentin, L1CAM or the transcription factor Zeb1 [6–8]. Furthermore, loss of cell differentiation may coincide with the acquisition of cancer stem cell (CSC)-characteristics of the tumor cells [9–11]. Mani *et al*. [9] were the first who demonstrated that breast cancer cells that have undergone EMT acquire a stem cell-like phenotype, and likewise, stem cell-like cells resemble cells that have undergone EMT.

CSCs are a small group of cancer cells within a cancer cell population with the unique ability to self-renew and to generate more differentiated cells. Thus, CSCs have been shown to be essential for tumor initiation, development, progression, recurrence and therapy resistance in various cancer entities, including PDAC [12–14]. Recent studies indicate that CSC-properties can be gained and lost depending on the microenvironment [11, 15–18], indicating that CSCs are not a stable cell population. Several markers, e.g. ABCG2, CD133, CD24 and CD44, have been proposed for CSCs in PDAC indicating a high heterogeneity and plasticity within the CSC population [19–21]. Besides the above mentioned markers, the transcription factor Nanog and the intermediate filament Nestin seem to play a role in the maintenance of CSC-properties [22, 23]. Moreover, it has been shown that Nestin impacts cell motility and EMT-properties in PDAC cell lines. Knockdown of Nestin in PDAC cells led to a reduced tumor incidence and volume as well as reduced formation of metastases in a murine PDAC model [24, 25]. One of the main metastatic sites of PDAC next to lung and peritoneum is the liver. While an impact of the liver microenvironment on EMT of prostate and breast cancer cells has been shown [26, 27], only few studies have addressed the role of the liver microenvironment on the development of PDAC metastases. Next to hepatocytes, hepatic stellate cells (HSCs) and their activated inflammatory counterpart, the hepatic myofibroblasts (HMFs), play an important role in liver tissue homeostasis, especially during injury and inflammation [28–31]. Furthermore, HMFs are also thought to be important for establishing a prometastatic microenvironment [29, 32]. A previous study of our group has provided evidence that HSCs contribute to induction and maintenance of a dormant phenotype in pancreatic ductal epithelial cells (PDECs) of different malignant potential. In contrast, HMFs have been shown to support the reversal of this dormant stage and foster metastatic outgrowth [33].

However, until now it is unclear how the hepatic microenvironment impacts on disseminated pancreatic ductal epithelial cells regarding their EMT- and CSC-characteristics. Thus, the present study aimed at analyzing the influence of different hepatic stromal conditions, displayed by hepatocytes and hepatic stellate cells or myofibroblasts, respectively, on cell growth, differentiation and self-renewal of PDECs by using an indirect coculture approach *in vitro* and a tumorigenicity PDAC mouse model *in vivo.*

## RESULTS

### The hepatic microenvironment supports self-renewal of premalignant and malignant PDECs

When disseminated PDECs settle in a secondary organ, they are exposed to a new microenvironment which impacts their growth behavior and survival [33]. In the liver, hepatocytes as well as hepatic stromal cells like HSCs and, upon inflammatory processes, HMFs play an important role in this scenario. Since it is still poorly understood how the hepatic microenvironment impacts CSC-features and differentiation of PDECs, we employed an indirect coculture system with different hepatic stromal cells and H6c7-kras cells (harboring only mutant *kras* as a PDAC-associated mutation) or malignant Panc1 cells, the latter exhibiting several PDAC-associated genetic and epigenetic alterations.

**Figure 1:**
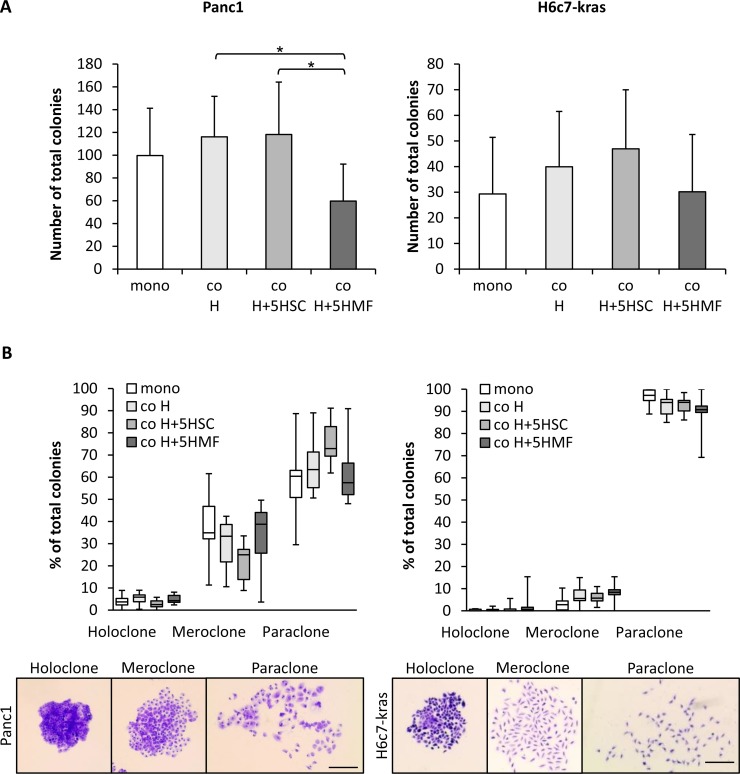
The hepatic microenvironment supports self-renewal of PDECs Panc1 and H6c7-kras cells were either monocultured (mono) or indirectly cocultured in different experimental hepatic environments, consisting of hepatocytes alone (co H) or hepatocytes enriched with 5% HSC (co H+5HSC) or 5% HMF (co H+5HMF), respectively, for 6 days. (**A**, **B**) After 6 day culture under the described conditions, PDECs were detached and 400 cells seeded for colony formation which was assessed after crystal violet staining on day 10. Only colonies containing more than 50 cells were counted and (A) the total number of colonies and (B) the proportion of different colony types of total number of colonies were determined. Due to reasons of clarity, significant differences are not marked in this chart. Data are presented as mean and standard deviation or median and quartiles (Q1 as 25% and Q3 as 75%) of 6 to 7 independent experiments. Below, representative images of crystal violet-stained holo-, mero- and paraclones of Panc1 and H6c7-kras cells are shown. Scale bar 250 μm. ^*^ indicates statistically significant differences (*p* ≤ 0.05).

In order to mimic a physiological microenvironment, coculture of either Panc1 or H6c7-kras cells was performed with hepatocytes alone (co H) or hepatocytes together with 5% HSC (co H+5HSC). For mimicking the inflamed liver micromilieu, Panc1 or H6c7-kras cells were cocultured with hepatocytes in the presence of 5% HMF (co H+5HMF). To study the influence of the different hepatic stromal conditions on self-renewal capacity of PDECs, colony formation assays (CFAs) were performed with Panc1 and H6c7-kras cells after 6 days of mono- or coculture. As expected, the ability to form colonies was higher in malignant Panc1 cells than in premalignant H6c7-kras cells independent of the culture conditions (Figure 1A). In both PDEC lines, colony formation was most pronounced after HSC-enriched coculture and lowest after coculture with hepatocytes and HMF (Figure 1A). Panc1 cells mostly formed paraclones which are supposed to be comprised of more differentiated cells, but they also gave rise to a considerable amount of mero- (25.0–38.8%) and holoclones (2.5–5.9%), the latter being supposed to contain the highest proportion of CSCs (Figure 1B). Thus, while the number of colonies of Panc1 cells was highest under HSC-enriched conditions, the formation of distinct colony types was not affected by the different hepatic stromal conditions. H6c7-kras cells also predominantly formed paraclones but in contrast to Panc1 cells, rarely formed meroclones (2.8–8.4%) or holoclones (0.0–0.6%) (Figure 1B). To confirm these findings, coculture experiments with human hepatocytes, hepatic stellate cells and hepatic myofibroblasts and either H6c7-kras, Panc1 cells or another PDAC cell line Panc89 were performed. In accordance with our findings described in Figure 1, malignant Panc1 and Panc89 cells formed more colonies than H6c7-kras cells and colony formation was highest in all three PDEC lines under coculture with human hepatocytes and 5% human hepatic stellate cells (Supplementary Figure 1). Overall, these data suggest that the self-renewal capacity of PDECs is maintained in the liver microenvironment even in an HSC enriched liver microenvironment resembling a physiological liver.

### Panc1 holoclones display enhanced formation of holoclones and Nestin expression compared to paraclones

**Figure 2:**
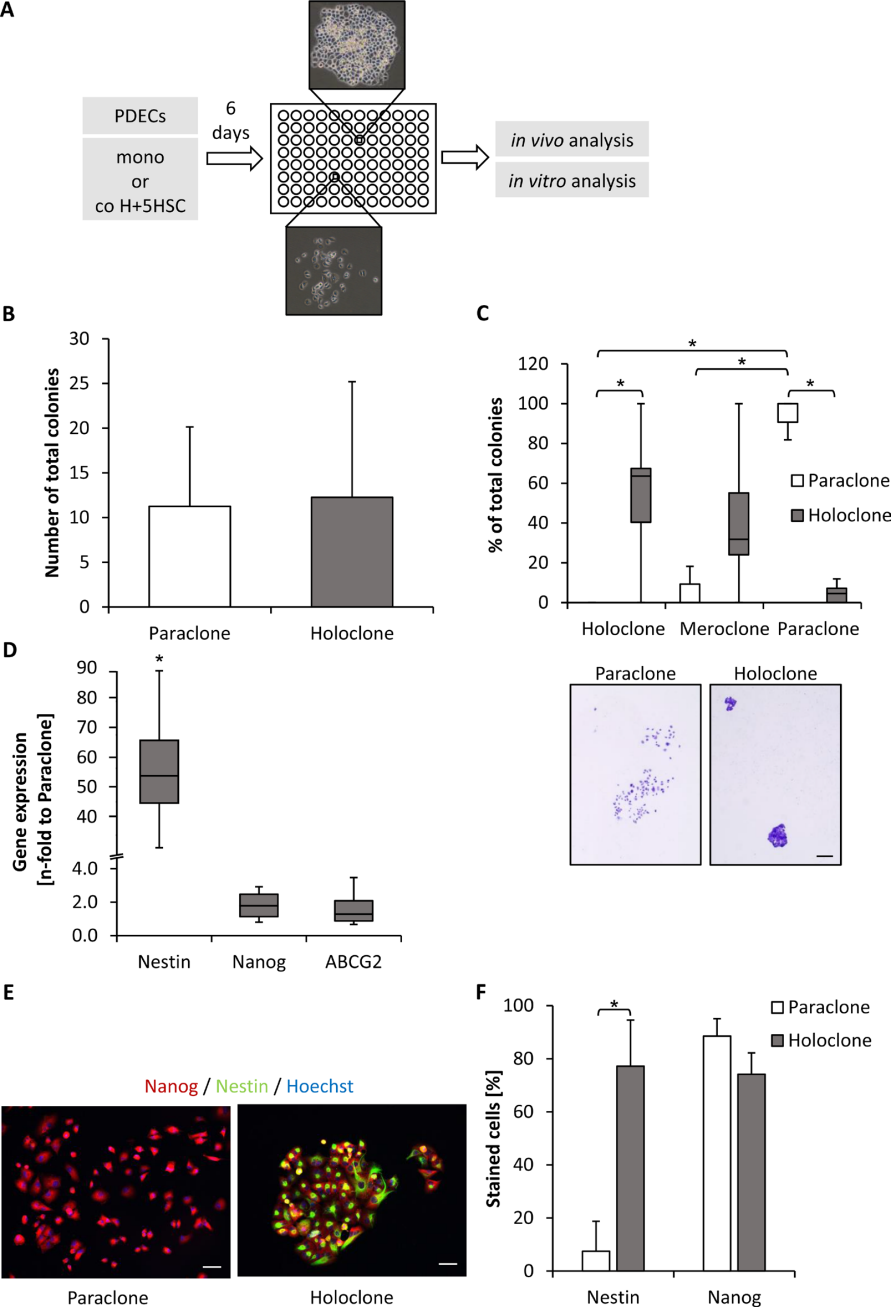
Panc1 holoclones display enhanced formation of holoclones and Nestin expression compared to paraclones (**A**) Scheme of generation and expansion procedure of different PDEC clones. After 6 day culture as depicted, single PDECs were seeded in a 96-well plate until single colonies were visible and their phenotype was assessed and confirmed by CFA. Only cells with stable phenotype were considered for further expansion and experiments. (**B**, **C**) In order to assess self-renewal capacity of expanded Panc1 holo- and paraclone cells, 400 cells were seeded for colony formation which was assessed after crystal violet staining on day 10. Only colonies containing more than 50 cells were counted and (B) the total number of colonies and (C) the proportion of different colony types of total number of colonies was determined. Data are presented as mean and standard deviation or median and quartiles (Q1 as 25% and Q3 as 75%) of 6 to 7 independent experiments. Below, representative images of crystal violet-stained Panc1 para- and holoclones are shown. Scale bar 250 μm. (**D**) RT-qPCR analysis of Nestin, Nanog and ABCG2 mRNA-expression normalized to GAPDH as housekeeper in Panc1 para- and holoclones. Data are presented as n-fold gene expression of paraclones and as median and quartiles (Q1 as 25% and Q3 as 75%) of 4 independent experiments. (**E**, **F**) Immunofluorescence staining of CSC-markers Nanog (red) and Nestin (green) in Panc1 paraclone and holoclone cells after *in vitro* expansion. Hoechst staining (blue) was performed to mark nuclei. Scale bar 25 μm. (F) Percentage of positively stained cells was determined by considering all cells contacting a horizontal line in 5 randomly chosen fields of view. Proportion of stained cells to total cell number is shown. Data are presented as mean and standard deviation of 3 independent experiments. ^*^ indicates statistically significant differences (*p* ≤ 0.05).

To characterize pancreatic CSCs that are able to survive and proliferate in HSC-enriched coculture conditions, a single cell dilution assay was performed with all three PDEC lines after coculture with H+5HSC and also after monoculture (Figure 2A). In parallel to the expression of different clone types, CFAs were regularly performed to confirm colony types and only cells with a robust phenotype were considered for further *in vitro* and *in vivo* experiments (Figure 2, Supplementary Figure 2). Clonal dilution and expansion of malignant Panc1 (Figure 2B, 2C) and Panc89 cells (Supplementary Figure 2) led to stable generation of para- and holoclones while in premalignant H6c7-kras cells only para- and meroclones could be stably established (Supplementary Figure 2). The total number of formed colonies was similar for both colony types obtained with Panc1 cells (Figure 2B). Moreover, Panc1 paraclone cells predominantly formed paraclones again and only rarely mero- and holoclones (Figure 2C). In contrast, H6c7-kras meroclones and Panc89 holoclones formed more colonies than the respective paraclone cells (Supplementary Figure 2A). H6c7-kras paraclone cells predominantly formed paraclones and only rarely mero- and holoclones while H6c7-kras meroclones also predominantly formed paraclones but concomitantly 32% meroclones (Supplementary Figure 2B). Panc89 holoclone cells predominantly formed holo- and meroclones (45.0% and 51.1%) along with 17% paraclones while paraclone cells formed mostly meroclones (62.9%) and lower numbers of holo- (21.1%) and paraclones (15.9%) (Supplementary Figure 2B). The latter observation might be explained by the fact that most isolated paraclones stopped proliferation during expansion and only those clones could be expanded containing a higher amount of holo- and meroclone cells. Having verified the differences in self-renewal capacity, cells from holo-/mero- and paraclones were further characterized with respect to CSC-marker expression. Holoclones from malignant PDECs showed elevated expression of Nestin, Nanog and ABCG2 of which Nestin was most strikingly elevated (Figure 2D, Supplementary Figure 2C). Premalignant H6c7-kras cells hardly forming any holoclones showed only significantly elevated Nanog expression (Supplementary Figure 2C). To verify these expression differences of the two CSC-markers on the protein level, immunofluorescence stainings were performed in Panc1 cells revealing considerable Nanog expression in both clone types, but with a higher number of strongly stained cells in paraclones (Figure 2E, 2F). In contrast, Nestin was exclusively expressed by holoclone-derived Panc1 cells (Figure 2E, 2F). Overall, these data demonstrate that premalignant H6c7-kras cells showed less pronounced self-renewal capacity indicated by the absence of established holoclone cells along with the absence of Nestin expression but pronounced Nanog expression. In contrast, holoclone cells of malignant Panc1 and Panc89 cells were characterized by pronounced self-renewal capacity and elevated Nestin expression.

### Panc1 holoclone cells derived from an HSC-enriched coculture exhibit an increased tumorigenic potential leading to formation of undifferentiated (mesenchymal) tumors

**Figure 3:**
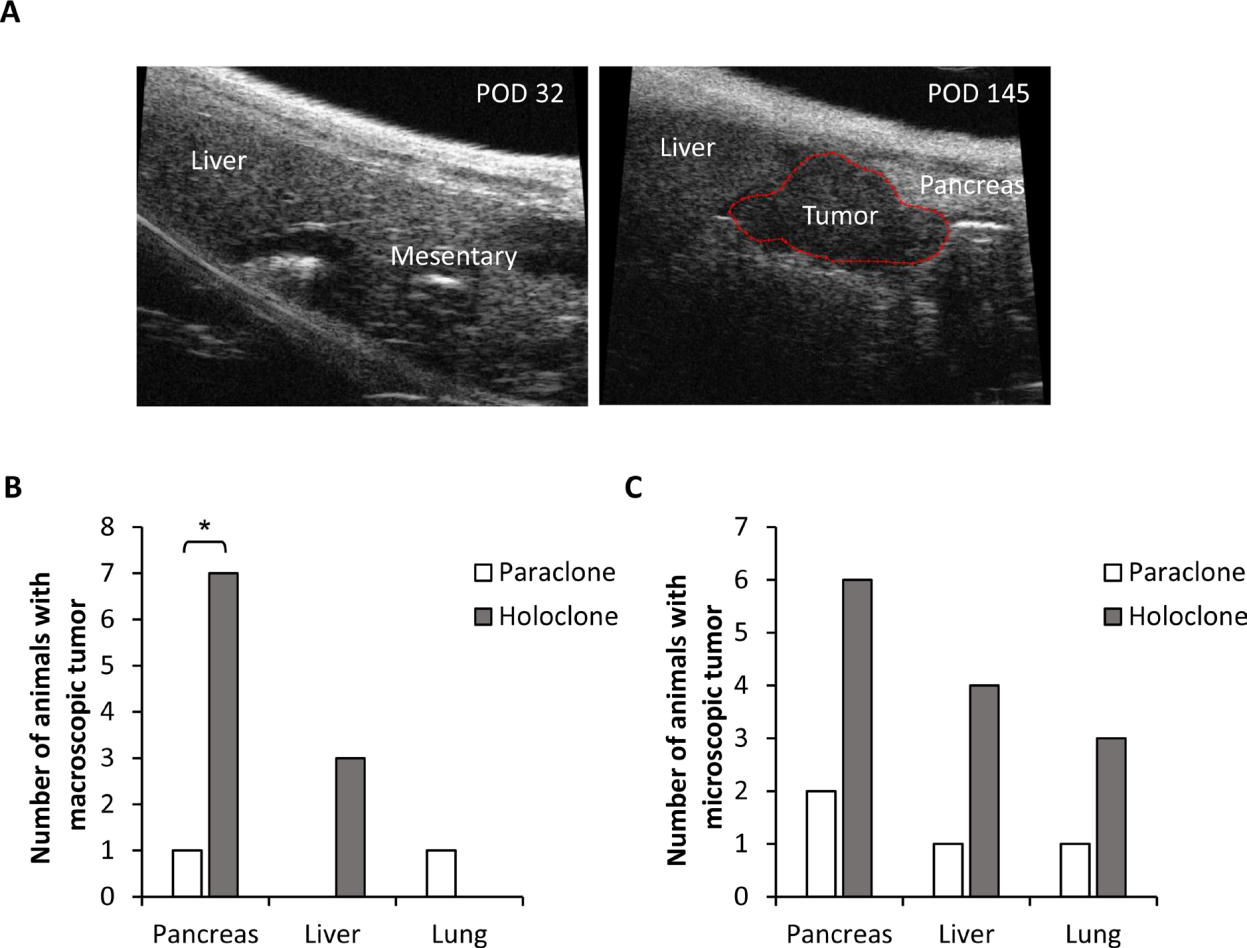
Panc1 holoclone cells exhibit an increased tumorigenic potential *in vivo* Tumorigenic potential of Panc1 holoclone and paraclone cells derived from an HSC-enriched coculture was investigated by intrasplenic inoculation of 10^4^ holoclone or paraclone cells into SCID-beige mice (*n* = 10 animals/cell type). (**A**) Representative sonographic images of an animal inoculated with Panc1 holoclone cells on day 32 and 145 post operation (POD). A macroscopic tumor lesion is encircled by red line (right panel). (**B**) Number of animals with macroscopic tumors in pancreas, liver and lung and (**C**) microscopic tumors in pancreas, liver and lung detected by immunofluorescence staining of human cytokeratin in formalin-fixed paraffin-embedded tissue sections of resected organs. ^*^ indicates statistically significant differences (*p* ≤ 0.05).

Next, it was investigated whether elevated self-renewal capacity of holoclones expanded from HSC-enriched coculture is associated with an enhanced tumorigenic potential *in vivo*. Since the phenotype of expanded Panc1 holo- and paraclone cells was most stable, 10^4^ holo- or paraclones-derived Panc1 cells were intrasplenically inoculated in SCID-beige mice and tumor formation was monitored by ultrasound over a period of almost 5 months (Figure 3A). While inoculation of 10^4^ Panc1 paraclone cells only led to macroscopic tumor formation in pancreas and lung in 1/10 animals each, Panc1 holoclone cells led to pancreatic tumor growth in 7/10 animals and formation of macroscopic liver metastases in 3/10 animals (Figure 3B). Detection of inoculated Panc1 cells by immunofluorescence staining targeting human cytokeratin revealed additional microscopic tumoral lesions in both holoclone- and paraclone-inoculated animals. However, more and larger microscopic lesions were detected in pancreas, liver and lung of animals inoculated with holoclone-derived cells (Figure 3C).

**Figure 4:**
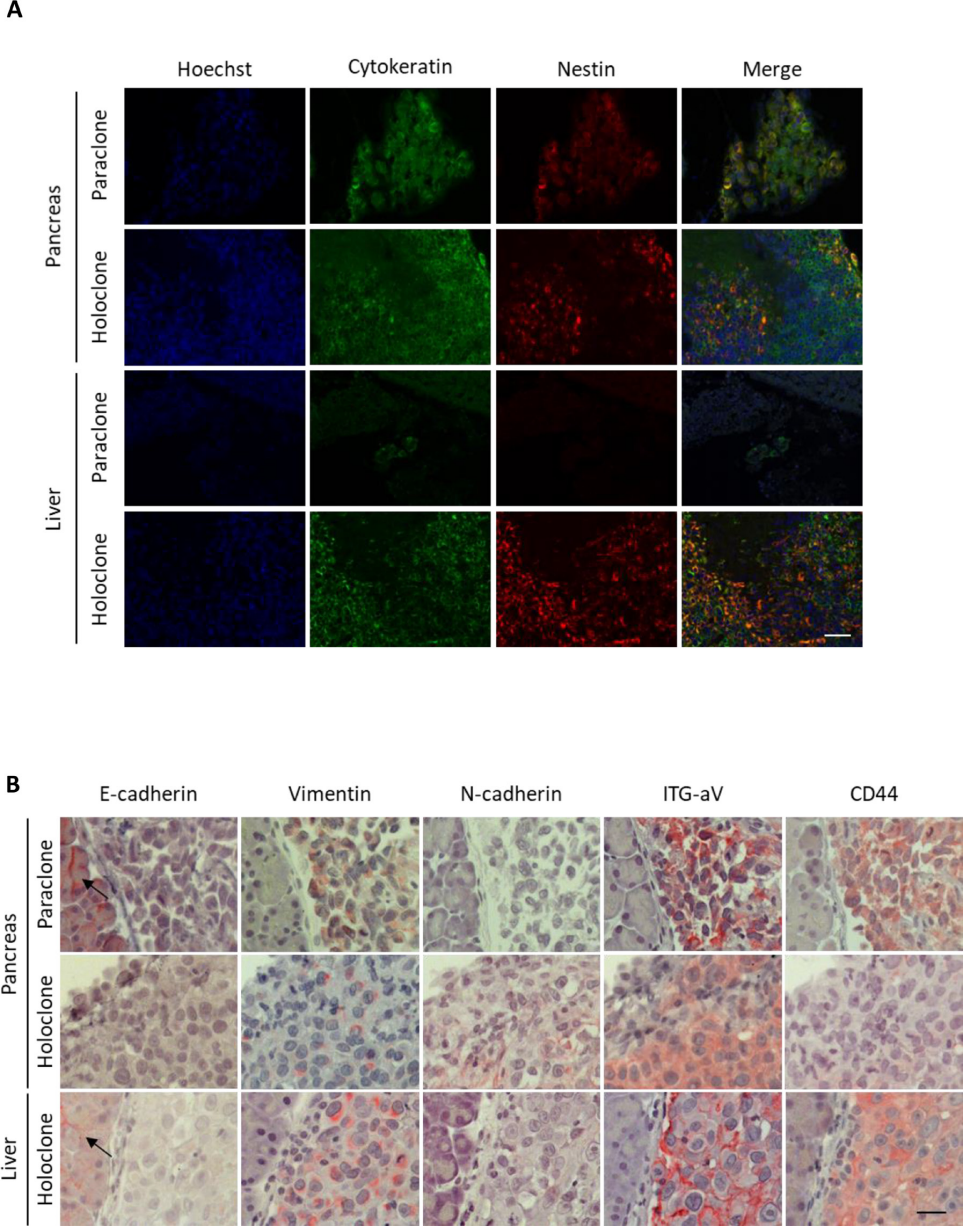
Panc1 holoclone tumors exhibit a pronounced mesenchymal phenotype along with elevated Nestin expression Tumorigenic potential of Panc1 holoclone and paraclone cells derived from an HSC-enriched coculture was investigated by intrasplenic inoculation of 10^4^ holoclone or paraclone cells into SCID-beige mice (*n* = 10 animals/cell type). Formalin-fixed paraffin-embedded tissue sections of pancreatic and corresponding liver sections were (**A**) immunofluorescently co-stained for human Cytokeratin (green) for detection of human Panc1 cells and the CSC-marker Nestin (red). Hoechst staining was performed to mark nuclei (blue). Scale bar 50 μm. (**B**) Pancreatic and hepatic tissues were immunohistochemically stained for E-cadherin, Vimentin, N-cadherin, Integrin alpha V (ITG-aV) and CD44. Arrows indicate E-cadherin-expressing non-neoplastic tissue cells. Haemalumn staining was performed to counterstain nuclei. Scale bar 25 μm.

In line with the *in vitro* data, holoclone-derived tumoral lesions exhibited strong expression of Nestin. Moreover, only the single paraclone-derived pancreatic tumor, but not the corresponding small hepatic tumoral lesion showed weak Nestin expression (Figure 4A).

Furthermore, both pancreatic and hepatic holoclone tumors displayed moderate to strong expression of the mesenchymal markers Vimentin, N-cadherin, Integrin alpha V as well as CSC-marker CD44 as detected in immunohistochemical stainings. In contrast, expression of epithelial E-cadherin was completely absent in tumor cells and only found in adjacent non-tumoral pancreatic or liver epithelial cells (Figure 4B). Due to the small size of the only hepatic lesion derived from paraclones, we could not determine EMT-marker expression in this tissue. However, in the paraclone-derived pancreatic tumor similar expression patterns of Vimentin, N-cadherin, Integrin alpha V, CD44 and E-cadherin could be detected as in holoclone tumors (Figure 4B). These *in vivo* data suggest that Panc1 holoclone cells derived from an HSC-enriched coculture exhibit an increased tumorigenic potential leading to formation of undifferentiated (mesenchymal) tumors predominantly in pancreas and liver.

### Panc1 holoclone cells exhibit a more pronounced mesenchymal phenotype than paraclones which is partly dependent on TNF-α

**Figure 5:**
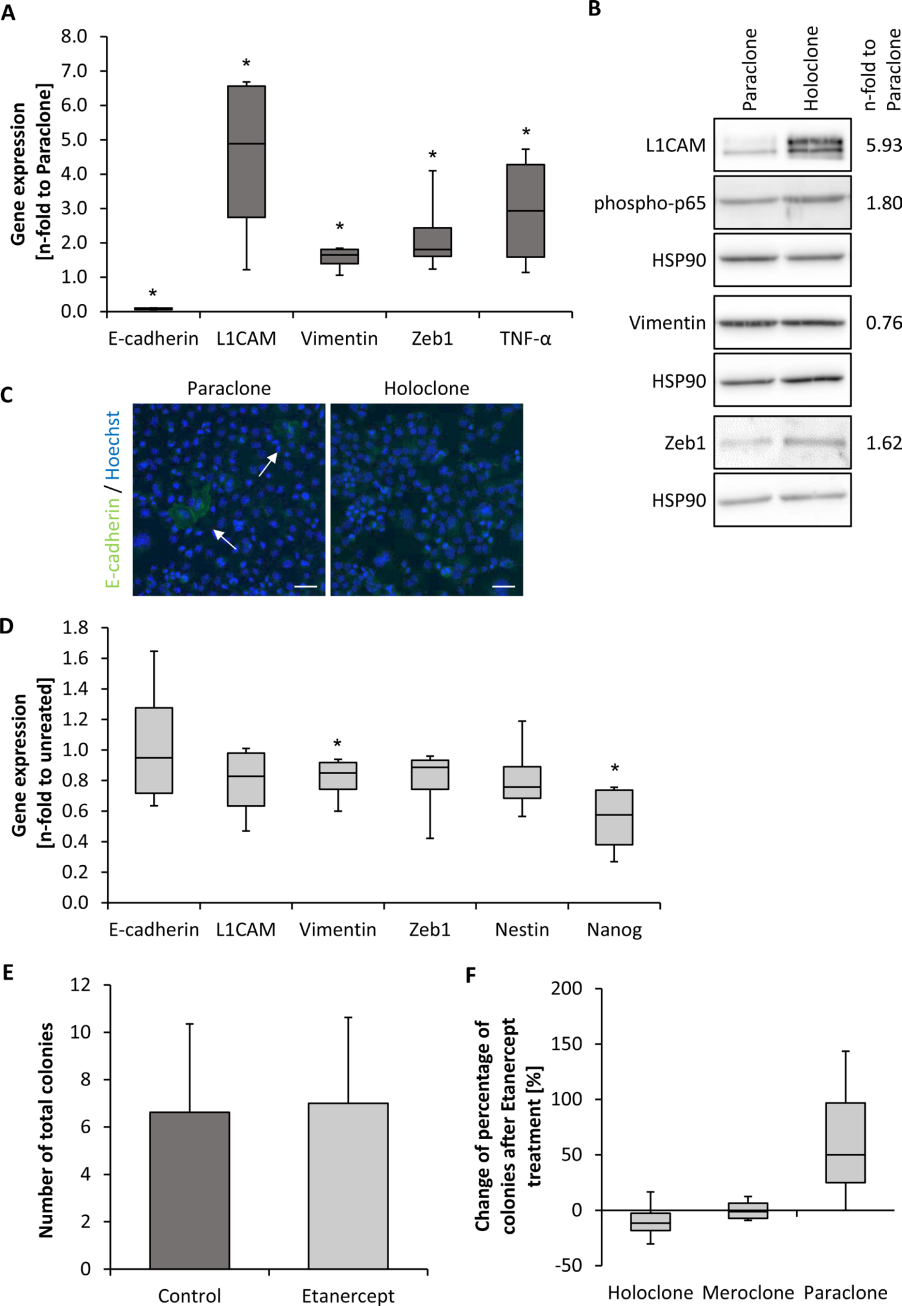
Panc1 holoclone cells exhibit a pronounced mesenchymal phenotype which is partly dependent on TNF-α signaling EMT-profile of Panc1 holo- and paraclone cells derived from expansion procedure. (**A**) RT-qPCR analysis for expression of E-cadherin, L1CAM, Vimentin, Zeb1 and TNF-α normalized to the housekeeping gene GAPDH and presented as n-fold expression compared to expression in paraclone cells. (**B**) Western blot analysis of L1CAM, Vimentin, phospho-p65 and Zeb1 in Panc1 para- and holoclone cells. HSP90 was used as loading control. One representative result of 4 independent experiments is shown. Numbers at the right side of the bands indicate average band intensities of holoclone probes determined by densitometric analysis. Values of the proteins of interest were divided by the values of the corresponding loading control (HSP90) and normalized to values of paraclones. (**C**) Immunofluorescence staining of E-cadherin (green) in Panc1 para- and holoclone cells. Hoechst staining (blue) was used for nuclei detection. Arrows indicate E-cadherin-expressing cells. Scale bar 50 μm. (**D**) RT-qPCR analysis for expression of E-cadherin, L1CAM, Vimentin, Zeb1 as well as Nestin and Nanog in Panc1 holoclone cells that were left untreated or treated with 10 μg/ml Etanercept for 72 hours. Specific gene expression was normalized to the housekeeping gene GAPDH and presented as n-fold expression compared to control. (**E**, **F**) For examining the influence of Etanercept on colony formation, 400 Panc1 holoclone cells were seeded in duplicates and treated with 10 μg/ml Etanercept on day 1 and 7 or left untreated as control. Colony formation was assessed after crystal violet staining on day 11. Only colonies containing more than 50 cells were counted and (E) the total number of colonies was determined as well as (F) the proportion of different colony types of total number of colonies. Data are shown as percentage change of Etanercept-treatment compared to control. Data are presented as mean and standard deviation or median and quartiles (Q1 as 25% and Q3 as 75%) of 4 independent experiments. ^*^ indicates statistically significant differences (*p* ≤ 0.05).

Since holoclone-derived tumors exhibited characteristics of EMT, Panc1 holo- and paraclone cells were characterized with respect to further molecular aspects of EMT. In line with our *in vivo* data, Panc1 holoclone cells exhibited a reduced E-cadherin expression and higher expression levels of L1CAM, Vimentin, Zeb1 and Tumor Necrosis Factor-alpha (TNF-α) compared to paraclones indicating a stronger mesenchymal phenotype (Figure 5A). Western blot analyses confirmed elevated L1CAM and Zeb1 expression in holoclones (Figure 5B). Moreover, a higher expression of the activated (phosphorylated) p65 subunit of NF-κB could be detected in Panc1 holoclone cells fitting together with the elevated TNF-α expression in these cells (Figure 5A, 5B). Due to low abundance of E-cadherin-expressing Panc1 cells, immunofluorescence staining was performed instead of western blot analysis revealing single E-cadherin positive cells within the paraclone cell population (Figure 5C). In contrast, no E-cadherin expressing cells were visible in holoclone-derived Panc1 cells (Figure 5C), further supporting the more pronounced mesenchymal phenotype of these cells. EMT profiling of H6c7-kras mero- and paraclone cells revealed that H6c7-kras meroclone cells exhibited considerable expression of mesenchymal proteins as observed in Panc1 holoclone cells. However, no reduction in E-cadherin expression could be detected indicating a mixed epithelial-/mesenchymal phenotype of H6c7-kras meroclone cells (Supplementary Figure 3A). In contrast, Panc89 holoclone cells showed significant reduction in E-cadherin expression but concomitantly either reduction or no difference in the expression of mesenchymal proteins compared to the paraclone cells (Supplementary Figure 3B) pointing to a mixed epithelial-/mesenchymal CSC-like phenotype. Overall, these findings indicate that acquisition of CSC-properties and an EMT phenotype occurs stepwise in PDECs and that premalignant and malignant PDECs are heterogenous with regard to EMT/CSC-properties. This also implies that PDECs with a high CSC-potential can differ with respect to their differentiation.

Since mesenchymal Panc1 holoclones (and also H6c7-kras meroclones) exhibited higher TNF-α expression, we next investigated whether this expression change might contribute to the maintenance of the more pronounced CSC-phenotype of holoclone-derived Panc1 cells. Therefore, Panc1 holoclone cells were treated either with Etanercept for 72 h to block TNF-α activity or cultured under comparable conditions in the absence of Etanercept as control. Blockade of TNF-α did not influence expression of E-cadherin, but reduced expression of the mesenchymal proteins L1CAM, Vimentin and Zeb1. In addition, expression of CSC-markers Nestin and predominantly Nanog was also reduced (Figure 5D). Although TNF-α blockade did not diminish the total number of formed colonies (Figure 5E), it considerably reduced formation of CSC-enriched holoclones in favor of paraclone formation, being consistent with the reduced CSC-marker expression (Figure 5F). These data suggest that the mesenchymal phenotype and CSC-properties of Panc1 holoclone cells are sustained by TNF-α indicating that this inflammatory cytokine is a common mediator of EMT and cancer stemness in mesenchymal PDAC cells.

## DISCUSSION

We have recently shown that the hepatic microenvironment represents an important determinant in induction and reversal of dormancy during PDAC metastases formation and that inflammatory alterations of the liver drive the outgrowth of PDAC metastases growth [33]. Importantly, self-renewal capacity as well as differentiation potential, both being sustained by CSCs, are mandatory for initiation and progression of tumors [13, 14, 34]. Therefore, this study aimed at extending the above mentioned findings by gaining a better understanding of the mechanisms by which the liver microenvironment impacts cancer stemness in PDAC metastases. For this purpose, we modeled the presence of disseminated PDECs as well as their heterogeneity in the hepatic microenvironment *in vitro* by coculturing H6c7-kras (epithelial-mesenchymal phenotype, harboring only kras mutation), Panc89 cells (moderately differentiated/ epithelial-mesenchymal phenotype, harboring several PDAC associated genetic alterations) and Panc1 cells (poorly differentiated/ mesenchymal phenotype, harboring several PDAC associated genetic alterations) either alone, in the presence of hepatocytes alone or together with 5% HSC (mimicking physiological conditions) or 5% HMF (mimicking inflamed conditions). As expected, colony formation of malignant Panc89 and Panc1 cells was more pronounced compared to H6c7-kras cells with incomplete malignant transformation. However, colony formation ability of all three PDEC lines was most pronounced in an HSC-enriched liver microenvironment which suggests that CSC-properties are well maintained and promoted by a physiological liver micromilieu. The observation that all PDEC lines formed fewer colonies after coculture under inflamed liver conditions (H+5% HMF) might be explained by the fact that HMFs promote PDEC proliferation [33] leading to dilution of the CSC pool. To further characterize the clone types formed under HSC-enriched conditions, holo-/meroclones and paraclones derived from single cocultured cells were expanded and analyzed. For PDAC, many CSC-markers have been proposed including Nestin and Nanog [22–24]. In contrast to a study by Skoda *et al*. [35], in which Nestin was expressed in 95% of all cells derived from primary PDAC cell lines, pointing to a common expression of this protein in the entire tumor cell population, we found Nestin to be differentially expressed in Panc1 and Panc89 para- and holoclones with Nestin being exclusively expressed in holoclone cells. Supporting the view that Nestin expression is a hallmark of cells with the highest self-renewal ability, meroclones formed by premalignant H6c7-kras cells hardly displayed any Nestin expression. Moreover, an increased Nestin expression correlated with enhanced formation of holoclones of Panc1 cells *in vitro* as well as an enhanced tumorigenicity *in vivo* leading to formation of Nestin-positive tumors in SCID-beige mice. These findings are consistent with other studies reporting that Nestin inhibition led to reduced cell migration and invasion *in vitro* as well as diminished metastases formation *in vivo* [12, 24]. In contrast to studies in prostate cancer, in which only inoculation of holoclones or meroclones resulted in tumor growth [36, 37], injection of Panc1 paraclone cells also led to tumor formation albeit in fewer animals (7/10 for holoclones, 2/10 for paraclones) as well as smaller in number and size compared to tumors formed by holoclone cells. Important to note, either clone cell population - even though expanded from a distinct colony type - was heterogeneous so that holoclone cells also formed paraclones and *vice versa* paraclones also contained single holoclone cells (see Figure 2B). Thus, formation of tumors by paraclone cells might be explained by the fact that holoclone cells, being present in the inoculated tumor cell population, led to scattered tumor formation. Alternatively, a certain number of disseminated paraclone cells may have regained CSC-/holoclone-properties owing to the influence of the microenvironment. This might also explain the Nestin expression detected in the single pancreatic paraclone lesion and, again, suggests that either the lesion originated from one of the few Nestin-positive holoclone cells within the inoculated paraclone cell population or Nestin expression was induced once the paraclone cells had settled in the pancreas. Interestingly, Nestin expression was not found in the hepatic paraclone lesion supporting the role of the microenvironment as a determining factor of cancer stemness and tumor formation [31, 38]. It has been shown that the transition between CSC- and non-CSC-states is linked to EMT and that its reversal, the Mesenchymal-Epithelial-Transition (MET), in cancer cells is highly dependent on soluble factors that e.g. might be released by the local environment [9, 10, 26]. Accordingly, Panc1 holoclone cells did not only exhibit high levels of Nestin expression, but also showed a pronounced mesenchymal phenotype associated with reduced expression of E-cadherin as well as increased expression of mesenchymal markers *in vivo* and *in vitro* indicating a mesenchymal phenotype with pronounced CSC-potential. H6c7-kras meroclone cells also exhibited enhanced expression of mesenchymal proteins compared to paraclones but concomitantly displayed E-cadherin expression pointing to a mixed epithelial-/mesenchymal phenotype along with limited CSC-potential. Finally, Panc89 holoclone cells showed marked formation of holoclones along with elevated Nestin expression compared to paraclone cells and exhibited reduced E-cadherin expression but no increased expression of mesenchymal proteins indicating a mixed epithelial-/mesenchymal phenotype but with pronounced CSC-potential. Overall, these data support the view that PDAC cells with a high CSC-potential (formation of holoclones *in vitro* and high tumorigenicity *in vivo*) do not necessarily have to exhibit a full mesenchymal phenotype (reduced expression of epithelial proteins and enhanced expression of mesenchymal proteins) in order to initiate tumor/metastases formation. It can be speculated that downregulation of E-cadherin is an essential step in increasing Nestin expression thereby conferring self-renewal capacity in PDAC cells as it has been shown in lung adenocarcinoma cells [39]. Known factors that promote EMT and CSC-properties are TNF-α or Transforming Growth Factor-beta1 (TGF-β1) [9, 10, 23, 40]. Indeed, the mesenchymal phenotype along with the CSC-characteristics in Panc1 holoclone cells could be partly reduced by blocking TNF-α, resulting in the formation of higher numbers of paraclones at the expense of holoclone abundance. These results further support the view of an interconnection between EMT-characteristics and CSC-potential in PDAC cells. In line with these findings, it could be shown that Nestin overexpression increases cell motility along with a more mesenchymal phenotype *in vitro*, whereas its downregulation favored an epithelial phenotype and reduced tumor incidence and size [12, 23, 41]. Owing to the fact that blockade of TNF-α did not completely inhibit colony formation (and with this self-renewal) of Panc1 cells points to other factors being involved in the regulation of differentiation and stemness properties in these cells. In this context it might be of interest that Panc1 cells secrete elevated levels of TGF-β1 [42, 43]. Since TGF-β1 expression did not significantly differ between holoclone and paraclone Panc1 cells, we did not further investigate its role in regulating holoclone and paraclone behavior of these cells. Krebs *et al.* [44] have recently identified Zeb1 as a major factor for the initiation of metastatic growth in pancreatic cancer with its absence being connected to reduced stemness and cellular plasticity [44, 45]. Enhanced expression of Zeb1 has also been associated with an inflamed microenvironment [46]. De Cock *et al*. [46] observed the outgrowth of lung metastasis from previously seeded latent disseminated tumor cells after induction of inflammation. This inflammatory-driven metastatic outgrowth was consistent with a causal activation of EMT being mediated by Zeb1 and Twist. In accordance with these findings, Zeb1 expression was significantly higher in Panc1 holoclone cells compared to paraclone cells and was also negatively affected by TNF-α blockage. Thus, these data also support a benefit for TNF-α blockade as therapeutic strategy for prevention or inhibition of PDAC primary and metastatic tumor growth. Egberts *et al*. [47] have already shown that TNF-α increases tumor growth and invasiveness of PDAC cells and that inhibition of TNF-α with Infliximab or Etanercept significantly reduces tumor burden in PDAC xenograft models. Moreover, TNF-α blockade performed after removal of primary tumors as adjuvant therapy efficiently diminished outgrowth of recurrent primary lesions as well as formation of liver metastases [47].

Overall, this study contributes to the understanding of the influence of the hepatic microenvironment on disseminated PDECs concerning CSC- and EMT-characteristics. Physiological conditions given by an HSC-enriched liver milieu efficiently regulate stemness and differentiation of PDECs. Together with former studies [33], these findings underscore the importance of the hepatic micromilieu in the outgrowth of liver metastases in PDAC.

## MATERIALS AND METHODS

### Ethics statement

All animal studies were executed in compliance with European guidelines for care and use of laboratory animals and approved by local authorities (V242-77780/2015 (123-10/11)).

### Cell lines and cell culture

All cell lines have recently been authenticated by using STR-analysis.

As a model for premalignant pancreatic ductal epithelial cells, the immortalized human PDEC line H6c7-kras containing a G12V-mutation in the *kras* gene (kindly provided by Prof. M.S. Tsao, Ontario Cancer Institute, Toronto, Canada) was used and grown in H6c7-medium (50% keratinocyte-serum free medium (Gibco Life Technologies, Darmstadt, Germany) and 50% Roswell Park Memorial Institute (RPMI) 1640 (Biochrom, Berlin, Germany) supplemented with 5% fetal calf serum (FCS), 0.5% L-Glutamine (both Biochrom), 50 μg/ml bovine pituitary extract, 5 ng/ml epidermal growth factor (both Gibco Life Technologies), and 0.5 μg/ml puromycin (Invitrogen, Darmstadt, Germany)). As a model for malignant and poorly differentiated PDECs, the human Panc1 cell line (purchased from ATCC) and as a model for moderately differentiated PDAC cells, the Panc89 cell line (kindly provided by Prof. T. Okabe, University of Tokyo, Japan) were used. The two PDAC cell lines were cultured in Panc-medium (RPMI 1640 supplemented with 10% FCS, 1% L-Glutamine and 1% sodium pyruvate (Biochrom)).

Murine hepatic stellate cells (M1-4 HSC, in the following termed HSC) were used to model a physiological liver microenvironment. Hepatic myofibroblasts (M-HT, in the following termed HMF) were generated from HSC by TGF-β1-exposition and were used to mimic an inflamed liver environment [28, 33]. Cells were grown in Mi-Medium (Dulbecco's Modified Eagle Medium (DMEM) supplemented with 4.5 g/l D-Glucose and 3.7 g/l NaHCO_3_ (Biochrom), 10% FCS, 1% sodium pyruvate and 1% L-Glutamine (Biochrom)). Detailed culture conditions have been described previously [33].

Primary murine hepatocytes were isolated as described [48] and grown in hepatocyte-medium (Williams’ Medium E (Biochrom) supplemented with 10% SeraPlus (PAN Biotech, Aidenbach, Germany), 1% L-Glutamine, 1% Penicillin/Streptomycin, 2 ng/μl Insulin-transferrin-sodium selenite supplement 100× (Sigma-Aldrich, St. Louis, USA), 10 μg/ml Gentamicin (Gibco Life Technologies), 100 nM dexamethasone (Sigma-Aldrich) on a collagen layer (Roche Diagnostics, Mannheim, Germany). Hepatocyte vitality varied between 77% and 98% when seeded for coculture experiments. Confirmatory experiments were performed with human hepatocytes (BioreclamationIVT (BIOIVT), West Sussex, UK; M00995-P LOT: MSW, IAN and AFJ), human HSC and human HMF. Human HSC were purchased from provitro AG (Berlin, Germany). Cells were taken into culture at passage 1 and cultured in SteCM Stellate Cell Medium (provitro AG). To generate a stable and reproducible phenotype of quiescent/weakly activated HSC or HMF human HSC were weekly treated with either 5 μM all−trans−Retinoic acid (Sigma Aldrich) to generate HSC or 1 ng/ml TGF-β1 (BioLegend, Koblenz, Germany) to generate HMF. After a time period of minimum 2 weeks, cells were set in coculture with either H6c7-kras, Panc1 cells or Panc89 cells under the same conditions as with MI1-4HSC or M-HT. Phenotypes of human HSC and HMF have been previously described [33].

### Coculture of PDECs and hepatic stromal cells

For indirect coculture experiments, all cells had been seeded and kept in their respective medium overnight, before cocultures were started by inserting ThinCert™ cell culture inserts (pore size 0.4 μm, Greiner Bio-One, Frickenhausen, Germany) containing PDECs into corresponding wells containing different hepatic stromal cells. Coculture was conducted for 6 days in a mixture of 50% Panc- and 50% Mi-medium. Monoculture of PDECs was performed under comparable conditions. Three hours after seeding 5 × 10^4^ murine or human hepatocytes on a collagen layer in a 6-well plate, cells were washed twice and medium was exchanged to SeraPlus-free hepatocyte-medium. Alternatively, 4.75 × 10^4^ murine or human hepatocytes were mixed with either 0.25 × 10^4^ murine or human HSC (H+5HSC) or HMF (H+5HMF) and were cultured in 50% SeraPlus-free hepatocyte-medium and 50% Mi-medium. One × 10^4^ PDECs were seeded in cell culture inserts in their respective medium.

### Colony formation assay (CFA)

CFAs were conducted with mono- and cocultured PDECs and clones derived from single cell expansion. Briefly, 400 single cells were seeded in duplicates into a 6-well culture plate in their respective medium. After cultivation for eight to eleven days, colonies were fixed with 4.5% paraformaldehyde for 10 min, stained with 0.1% crystal violet (Merck Millipore, Darmstadt, Germany) for 1 h, washed in water and air-dried. Only colonies containing more than 50 cells were counted and their morphology regarding holo-, mero- and paraclones was assessed [49, 50]. While holoclones consisted of tightly and homogenously clustered cells with a regular borderline, paraclones displayed dispersed and larger cells with an irregular borderline [50]. Meroclones exhibited an intermediate colony morphology [50].

### Single cell cloning and clone expansion

PDECs were harvested with trypsin, either directly from a culture flask or after 6 day-coculture with hepatocytes and 5% HSC (H+5HSC), to generate a cell suspension with 100 cells in 20 ml of the respective cell medium. 200 μl of this cell suspension were filled into a flat-bottom 96-well plate (Sarstedt, Nuembrecht, Germany) to seed one single cell per well. Three hours after seeding, first detection was performed by scanning the plate in the brightfield channel of a NyONE cell imager (SynenTec GmbH, Elmshorn, Germany). Only wells containing exactly one cell were considered for further analysis and experiments. After 12 days of regular observation, colony types were characterized and monitored until cell confluence. Only clones forming the same morphology of colonies in CFA were considered for further expansion, *in vitro* and *in vivo* experiments. As cell clones were only used for a maximum of 12 passages, cell clones were also expanded from monoculture conditions showing no differences to clones isolated from H+5HSC-enriched coculture (data not shown).

### Neutralization of TNF-α by etanercept

For determining the effects of TNF-α on EMT and stemness, 2.5 × 10^5^ (for RNA isolation) or 400 (for CFA) Panc1 holoclone cells were seeded in Panc-medium in 6-well plates and allowed to attach overnight. Ten μg/ml Etanercept (Pfizer Deutschland, Berlin, Germany) were applied for 72 h before total RNA was retrieved. Control cells were cultured under comparable conditions without the addition of Etanercept. For CFAs, colonies were grown for 11 days with a second addition of 10 μg/ml Etanercept on day 7.

### RNA isolation and RT-qPCR

Total RNA was isolated using the total RNA kit peqGOLD (PeqLab, Erlangen, Germany) and subjected to reverse transcription according to manufacturer's instructions (Fermentas via Thermo Fisher Scientific, Darmstadt, Germany). qPCR analysis was performed as duplicate analysis on a LightCycler 480 (Roche) including melting curve analysis as quality control. Primers (Eurofins, Ebersberg Germany; RealTime Primers via Biomol, Hamburg, Germany), primer sequences and annealing temperatures are listed in Table 1.

**Table 1:** Primers, their sequences and annealing temperatures used for RT-qPCR

Primer		Primer sequence (5′-3′)	Annealing temperature (°C)	Manufacturer
**E-cadherin**	forward	TGCTCTTGCTGTTTCTTCGG	55	RealTimePrimers
	reverse	TGCCCCATTCGTTCAAGTAG		
**GAPDH**	forward	TCCATGACAACTTTGGTATCGTGG	58	Eurofins
	reverse	GACGCCTGCTTCACCACCTTCT		
**L1CAM**	forward	GAACTGGATGTGGTGGAGAG	58	RealTimePrimers
	reverse	GAGGGTGGTAGAGGTCTGGT		
**Nanog**	forward	ACCTACCTACCCCAGCCTTT	58	RealTimePrimers
	reverse	CATGCAGGACTGCAGAGATT		
**Nestin**	forward	GAAACAGCCATAGAGGGCAAA	58	Eurofins
	reverse	TGGTTTTCCAGAGTCTTCAGTGA		
**TNF-α**	forward	TCCTTCAGACACCCTCAACC	58	Eurofins
	reverse	AGGCCCCAGTTTGAATTCTT		
**Vimentin**	forward	TCCAAGTTTGCTGACCTCTC	58	RealTimePrimers
	reverse	TCAACGGCAAAGTTCTCTTC		
**Zeb1**	forward	TCCATGCTTAAGAGCGCTAGCT	61	Eurofins
	reverse	ACCGTAGTTGAGTAGGTGTATGCCA		

### Western blot

Preparation of whole cell lysates and western blot analysis were performed as previously described [51]. The following primary antibodies were used at 1:1000 dilution: anti-E-cadherin (32A8; Cell Signaling, Frankfurt, Germany), anti-HSP 90α/β (F-8; Santa Cruz, Heidelberg, Germany), anti-L1CAM (9.3; kindly provided by Prof. G. Moldenhauer, German Cancer Research Center, Heidelberg, Germany), anti-Zeb1 (Novus Biologicals, Wiesbaden-Nordenstadt, Germany). Anti-Vimentin (V9; Santa Cruz) was applied 1:200 and anti-phospho-p65 (12H11; Merck Millipore) 1:500. HSP90 was used as loading control. Secondary antibodies anti-mouse IgG (HRP-linked) and anti-rabbit IgG (HRP-linked) (both Cell Signaling) were used at 1:2000 dilution. Primary antibodies were incubated at 4°C overnight, while secondary antibody incubation was performed for 1 h at room temperature (RT). Blots were incubated in Clarity Western ECL Substrate (Bio-Rad Laboratories, München, Germany) and visualization of proteins was performed by using the Fusion SL detection system (Vilber Lourmat, Eberhardzell, Germany).

### Immunofluorescence staining of cells grown on cover slips

Cells grown confluent on cover slips were fixed in 4.5% paraformaldehyde for 10 min and permeabilized with methanol for 10 min at −20°C. Blocking was performed using 4% Bovine Serum Albumin (BSA, Serva Electrophoresis, Heidelberg, Germany) in PBS supplemented with 0.3% Triton X-100 (Sigma-Aldrich) for 1 h at RT, before primary antibody incubation was performed at 4°C in a humidified chamber overnight. Antibodies were diluted in 1% BSA/PBS/0.3% Triton X-100 as follows: anti-Nanog (D73G4; Cell Signaling) 1:100, anti-Nestin (10C2; Thermo Fisher Scientific) 1:200 and anti-E-cadherin (32A8; Cell Signaling) 1:50. After washing, incubation with fluorochrome-labeled secondary antibodies (AlexaFluor 488 anti-mouse; AlexaFluor 546 anti-rabbit (both Life Technologies, Carlsbad, USA), diluted 1:500 in 1% BSA/PBS/0.3% Triton) and 2 μg/ml Hoechst 33258 (Sigma-Aldrich) for nuclear staining was performed for 1 hour at RT before samples were mounted in FluorSave Reagent (Merck Millipore) mounting medium. Control staining was performed in parallel with isotype control antibodies (Isotype control mouse IgG1 (MAB002; R&D Systems, Minneapolis, USA); Isotype control rabbit IgG (SP137; abcam, UK)) and showed no or only weak staining.

### Tumorigenicity assay *in vivo*

All animal studies were executed in compliance with European guidelines for care and use of laboratory animals and approved by local authorities (V242-77780/2015 (123-10/11)).

One × 10^4^ Panc1 holoclone or paraclone cells, derived from single cell expansion after HSC-enriched coculture and diluted in 75 μl PBS, were intrasplenically inoculated in 8-week old, female SCID-beige mice (each group *n* = 10) (Charles River, Sulzfeld, Germany). Progression of tumor formation was monitored regularly by palpation and abdominal ultrasound examination using Vevo 770 (FUJIFILM VisualSonics Inc., Toronto, Canada). After 145 days, mice were sacrificed and pancreata, livers and lungs were fixed in 4.5% PBS-buffered formalin and embedded in paraffin before sectioning (2–3 μm) and immunohistological examination.

### Immunofluorescence and immunohistochemical stainings of paraffin-embedded tissue sections

Formalin-fixed, paraffin embedded tissue sections were deparaffinized and rehydrated by xylene and decreasing alcohol concentrations. For immunofluorescence stainings, antigen retrieval was performed with citrate buffer pH 6.0 at sub-boiling temperature for 20 min before blocking, antibody incubation and mounting were performed as described above. Additionally, Sudan Black B treatment (0.1% in 70% Ethanol, Sigma-Aldrich) was performed for 20 min at RT preceding secondary antibody incubation. For immunohistochemical stainings, primary antibody anti-human Cytokeratin (Immunotec, Prague, Czech Republic) was applied 1:100. Anti-human/murine E-cadherin antibody was purchased from Dako-Agilent (Santa Clara, USA) and diluted 1:200 in Dako Antibody Diluent M3612. In preparation for antigen retrieval of immunohistochemical stainings, sections were heated overnight in Dako Antigen Retrieval Solution S 1699 in an 85°C water bath. Antigen retrieval for N-cadherin immunohistochemistry was achieved by treating the sections with Fast Enzyme (Zytomed, Berlin, Germany) for 5 min at room temperature. The anti-human N-cadherin antibody 18203 (abcam, Cambridge, UK) was used in a 1:50 dilution. Antigen retrieval for integrin alpha V was achieved by boiling the sections for 10 min in Dako Antigen Retrieval Solution S 1699 and treating them with an Integrin alpha V antibody (abcam # ab150361). For Vimentin immunohistochemistry, antigen retrieval was achieved by treating the sections for 20 min in a Dako S2367 pH9 antigen retrieval solution. The anti-Vimentin antibody (Dako M7020) was diluted 1:232 in Dako antibody diluent. CD44 was detected after antigen retrieval at 80 C for 10 min in a citrate buffer pH6 and application of the anti-CD44 antibody (BD Pharmingen 550392, Heidelberg, Germany) at a dilution of 1:100 in Dako antibody diluent. The antigen binding sites were detected by using ABC streptavidin complexes with alkaline phosphatase as the marker enzyme (Vectorlabs, Burlingame, USA).

### Statistical analysis

Statistical analysis was performed using SigmaPlot 12.5 (Systat, Erkrath, Germany). When comparing two groups with parametric datasets, paired *t*-tests were executed. For multiple groups with parametric data sets, repeated measures analysis of variance (one-way RM ANOVA) test was performed, while non-parametric data were analyzed with the Kruskal-Wallis one-way ANOVA on ranks test. Statistical significances were detected by Tukey test or Student-Newman-Keuls method with *p*-values beneath 0.05 regarded as statistically significant and marked by asterisks (^*^).

## SUPPLEMENTARY MATERIALS FIGURES



## Abbreviations

CFA: Colony formation assay
CSC: Cancer stem cell
EMT: Epithelial-Mesenchymal-Transition
H: Hepatocyte
HMF: M-HT
HMFs: Hepatic myofibroblasts
HSC: M1-4 HSC
HSCs: Hepatic stellate cells
MET: Mesenchymal-Epithelial-Transition
PDAC: Pancreatic ductal adenocarcinoma
PDECs: Pancreatic ductal epithelial cells
