# Supplementary material for: Liver metastasis of pancreatic cancer: the hepatic microenvironment impacts differentiation and self-renewal capacity of pancreatic ductal epithelial cells
Source: Oncotarget. 2018 Aug 3;9(60):31771–86. doi: 10.18632/oncotarget.25884 (PMC6114965; doi:10.18632/oncotarget.25884)
Supplement: Supplementary file 1 [file oncotarget-09-31771-s001.pdf]

Liver metastasis of pancreatic cancer: the hepatic microenvironment impacts differentiation and self-renewal capacity of pancreatic ductal epithelial cells

SUPPLEMENTARY MATERIALS

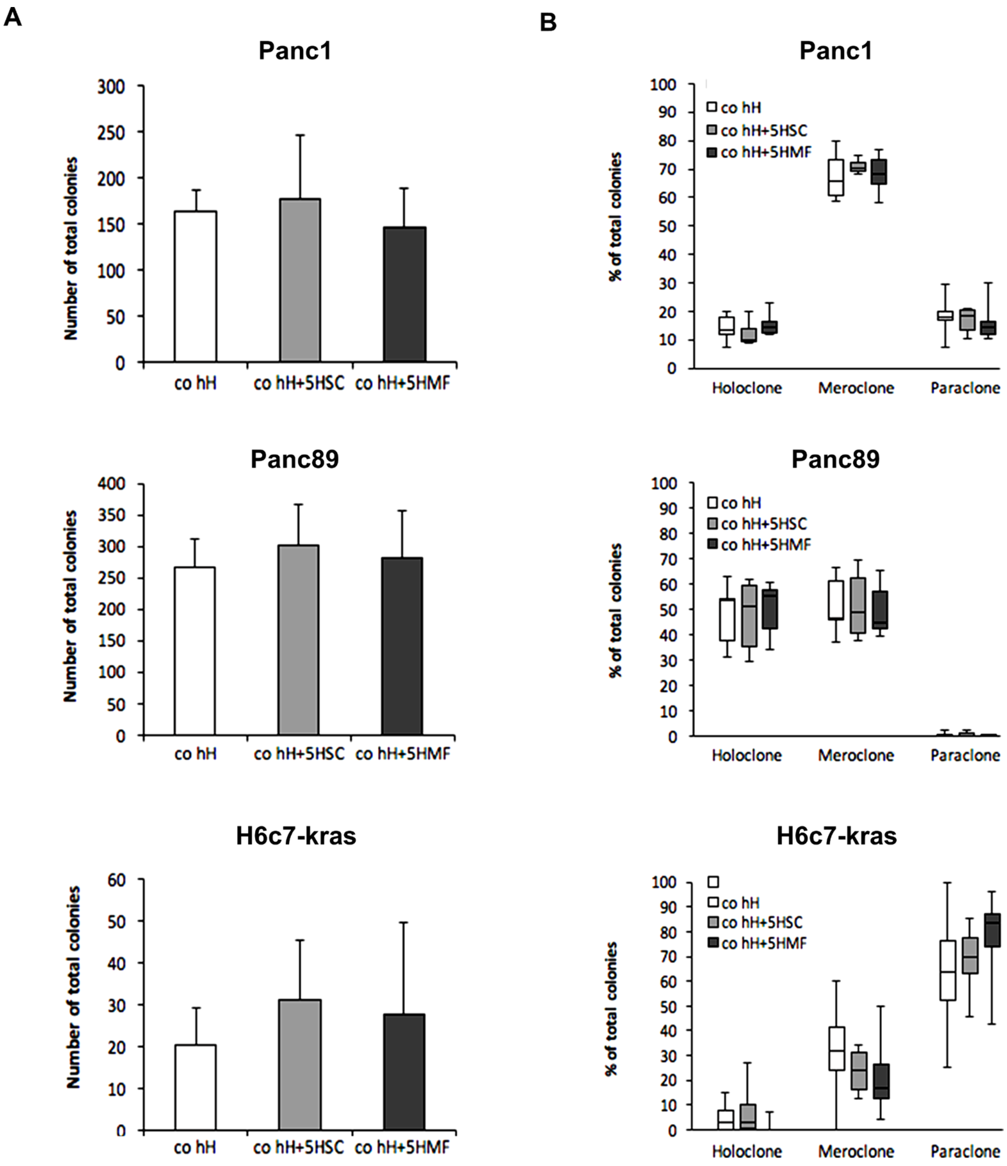

Supplementary Figure 1: The hepatic microenvironment supports self-renewal of PDEC. Panc1, Panc89 and H6c7-kras cells were indirectly cocultured in different experimental hepatic environments, consisting of human hepatocytes alone (co hH) or hepatocytes enriched with 5% human HSC (co hH+5HSC) or 5% human HMF (co hH+5HMF), respectively, for 6 days. (**A**, **B**) After 6 day culture under the described conditions, PDECs were detached and 400 cells seeded for colony formation which was assessed after crystal violet staining on day 10. Only colonies containing more than 50 cells were counted and (A) the total number of colonies and (B) the proportion of different colony types of total number of colonies were determined. Data are presented as mean and standard deviation or median and quartiles (Q1 as 25% and Q3 as 75%) of 3 independent experiments.

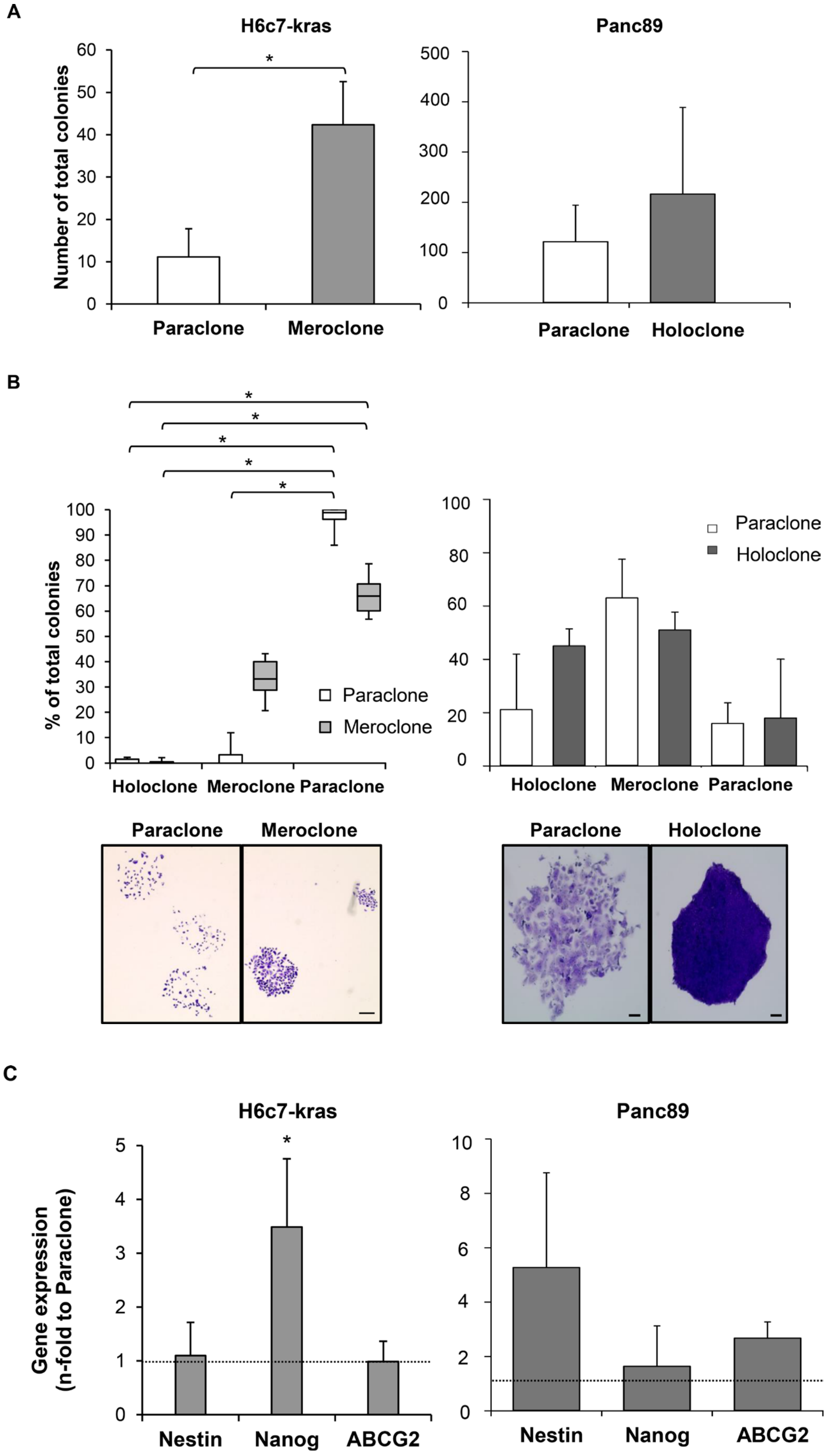

Supplementary Figure 2: Characterization of CSC-properties in H6c7-kras para- and meroclones as well as Panc89 para- and holoclones. In order to assess self-renewal capacity of clonogenically expanded H6c7-kras and Panc89 cells, 400 cells were seeded for colony formation which was assessed after crystal violet staining on day 10. Only colonies containing more than 50 cells were counted and (**A**) the total number of colonies and (**B**) the proportion of different colony types of total number of colonies was determined. Data are presented as mean and standard deviation or median and quartiles (Q1 as 25% and Q3 as 75%) of 3 independent experiments. Below, representative images of crystal violet-stained H6c7-kras para- and meroclones as well as Panc89 para- and holoclones are shown. Scale bar 250 µm (H6c7-kras) and 100 µm (Panc89). (**C**) RT-qPCR analysis of Nestin, Nanog and ABCG2 mRNA-expression normalized to GAPDH as housekeeper in H6c7-kras para- and meroclones as well as Panc89 para- and holoclones. Data are presented as n-fold gene expression of paraclones and as mean and standard deviation of 3 independent experiments. *indicates statistically significant differences ($p \leq 0.05$).

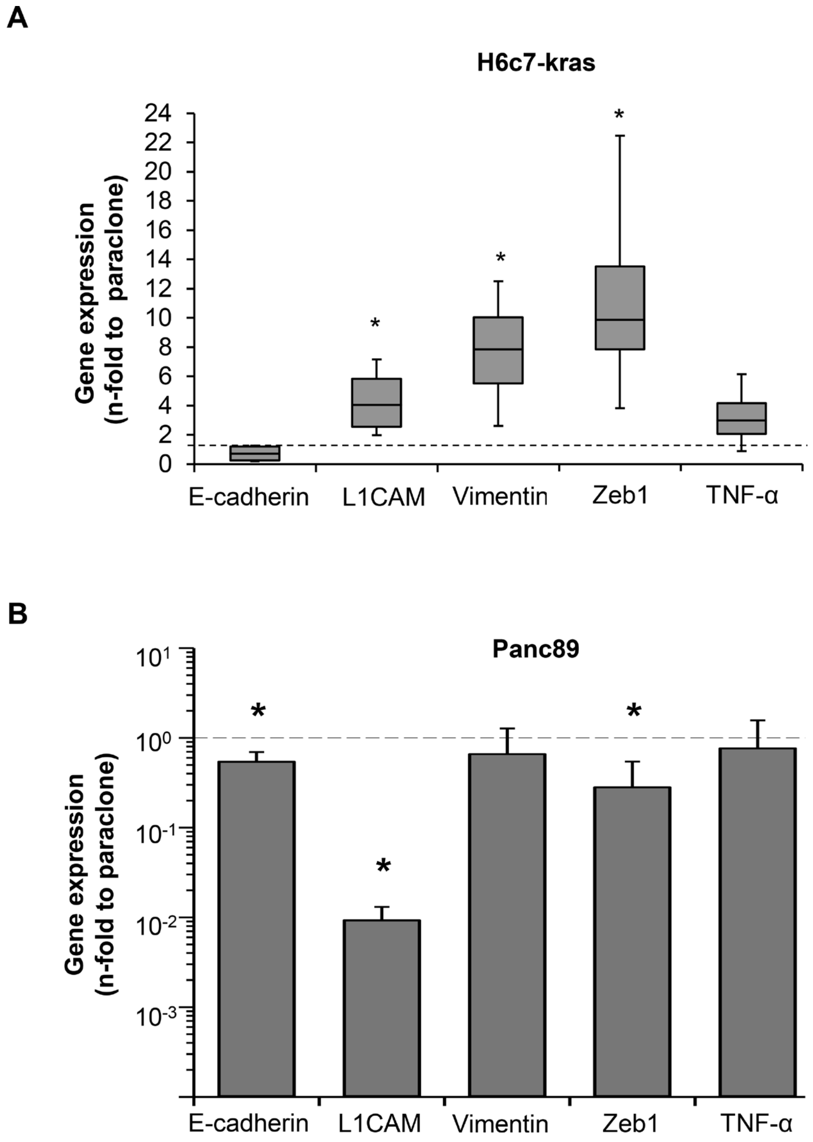

Supplementary Figure 3: Characterization of EMT-properties in H6c7-kras para- and meroclones as well as Panc89 para- and holoclones. EMT-profile of (**A**) H6c7-kras mero- and paraclone cells and (**B**) Panc89 holo- and paraclone cells derived from clonogenic expansion procedure was determined by RT-qPCR analysis for expression of E-cadherin, L1CAM, Vimentin, Zeb1 and TNF-α. Expression of either gene was normalized to the housekeeping gene GAPDH and presented as n-fold expression compared to expression in paraclone cells. Data are presented as mean and standard deviation or median and quartiles (Q1 as 25% and Q3 as 75%) of at least 3 independent experiments. * indicates statistically significant differences ($p \leq 0.05$).
